# Social media and data privacy in education: an international comparative study of perceptions among pre-service teachers

**DOI:** 10.1007/s40692-022-00243-x

**Published:** 2022-09-28

**Authors:** Victoria I. Marín, Jeffrey P. Carpenter, Gemma Tur, Sandra Williamson-Leadley

**Affiliations:** 1 Department of Pedagogy, University of Lleida, Lleida, Spain; 2 School of Education, Elon University, Elon, USA; 3 Department of Applied Pedagogy and Educational Psychology, University of the Balearic Islands, Ibiza, Spain; 4 School of Teacher Education, University of Canterbury, Christchurch, New Zealand

**Keywords:** Social media, Cultural implications, Comparative study, Data privacy, Higher education, Pre-service teacher education

## Abstract

Social media platforms offer many educational possibilities, but they also create challenges associated with their business models. One increasingly relevant challenge, especially in the context of teacher education and schools, is personal data privacy. When considering social media and data privacy in education, taking into account culture-specific aspects in different countries, such as legal frameworks, user attitudes, and cultural values, is uncommon. This cross-sectional study explores the perceptions of pre-service teachers (*N* = 225) from universities in four countries (Germany, New Zealand, Spain, and the USA) concerning educational and professional social media use, as well as data privacy awareness and practices. Data were collected via a survey and analyzed through descriptive and inferential statistics. Results indicate that along with common belief in social media’s educational potential, data privacy concerns were present, knowledge related to data privacy was lacking, and differences existed between participants from the different universities. We discuss these results in relation to legal frameworks, user attitudes, and cultural values concerning social media data privacy, and consider implications for research, practice, and policy.

**Supplementary Information:**

The online version contains supplementary material available at 10.1007/s40692-022-00243-x.

## Introduction

Social media platforms have permeated many facets of modern life, including education, where they have found increasingly widespread use for teaching and learning (Awidia et al., 2019; Greenhow et al., 2020). Social media have been used to extend learning activities by facilitating information access, communication, and networking (Lemon & O’Brien, 2019), and have been credited with providing support to students’ critical thinking, motivation, engagement, satisfaction, and even academic performance (Hidayati et al., 2021; Karakoyun & Lindberg, 2021). Educators may also potentially serve as role models for wise social media use for students (Vartiainen et al., 2022). In the field of teacher education, participation in social media activities in formal contexts can help pre-service teachers (PSTs) to learn about professional communities and resources to support their transition to in-service teaching (Kearney et al., 2020).

Similar to its mixed impact in other domains, the use of social media in education presents both benefits and challenges for educators and learners. One of the challenges, which has received relatively limited attention from researchers is *datafication*, a trend that involves human actions and interactions being turned into data that can be monitored, measured, analyzed, commodified, and used to inform decision-making (Cukier & Mayer-Schoenberger, 2013; Williamson et al., 2020). Education in particular is a sector where datafication can happen at many levels and has a variety of potential salutary and harmful effects (Jarke & Breiter, 2019; Krueger & Moore, 2015). Data-related matters have become even more important in light of the rapid shift to digital learning experienced by many schools and universities worldwide with the COVID-19 pandemic (Hodges et al., 2020).

Future teachers will likely need to teach about, with, and against social media (Krutka et al., 2019), while also attending to related matters of data privacy for themselves and their students (Milton et al., 2021; Vartiainen et al., 2022). Data privacy relates to the rights of citizens to control how their personal information is collected and used. In the social media context, data privacy is an issue of concern for Internet users world-wide (e.g., Auxier et al., 2019; klicksafe, 2018). However, how PSTs position themselves regarding social media data privacy may vary according to cultural differences (Trepte et al., 2017), which in turn relate to legal frameworks, users’ attitudes, and cultural values (Ur & Wang, 2013). Also, international and comparative research shows that PSTs from different countries may use and perceive social media differently (Carpenter et al., 2016, 2020; Karakoyun & Lindberg, 2021).

As the amount of personal data collected and shared by popular social media services increases, educators’ concerns and perceptions regarding data privacy issues should be carefully considered as potential influences on the use of social media in education (Ertmer & Ottenbreit-Leftwich, 2013; Prestridge, 2010; Teo, 2009). In this study, our objective is to explore the perspectives of PSTs from universities in four countries (Germany, New Zealand, Spain, and the USA) regarding professional and educational uses of social media and related data privacy issues. Our overarching research question is: What are the relationships between PSTs’ beliefs regarding professional and educational use of social media, and data privacy in social media? We divided this broad question into four sub-questions:

- RQ1. What are PSTs’ beliefs about educational and professional use of social media?
- RQ2. What are the differences in the perceptions of PSTs from universities in four countries concerning the educational and professional use of social media?
- RQ3. What are PSTs’ beliefs about and awareness concerning data privacy matters with social media services in educational contexts?
- RQ4. What are the differences between PSTs from universities in four countries’ beliefs about and awareness concerning data privacy matters with social media services in educational contexts?

Although some recent studies address related matters, we aim to provide further insights into a topic that is still in its infancy. Our research focuses on the understudied areas of educational use of social media by PSTs, and data privacy policies in the context of education (Milton et al., 2021; Vartiainen et al., 2022), includes cross-cultural comparisons between PSTs from different countries and areas than previous studies (Milton et al., 2021; Vartiainen et al., 2022), and features a bigger sample for quantitative analysis than prior qualitative studies (Prestridge et al., 2021; Vartiainen et al., 2022). In addition, we contribute to the literature by discussing the findings in light of a comprehensive framework that considers legal frameworks, user attitudes, and cultural values.

## Literature review

### Social media for teaching–learning processes in pre-service teacher education

Social media platforms appear to have affordances for supporting active learning and the development of twenty-first century skills. They have seen increasingly widespread use for teaching and learning (Awidia et al., 2019; Greenhow et al., 2020) due to their ease-of-use, accessibility, and effectiveness (Hidayati & Yuniati, 2021). Social media have been used to extend learning activities by facilitating information access, communication, and networking (Lemon & O’Brien, 2019), which could support language learning, critical thinking, motivation, engagement, satisfaction, and eventually improve students’ performance (Hidayati et al., 2021; Karakoyun & Lindberg, 2021).

The participation of PSTs in social media activities in formal contexts can help them to feel part of the larger education profession and learn about professional communities and resources that can support their transition to in-service teaching (Kearney et al., 2020). Furthermore, research has related the affordances of social media participation and collaboration with greater interactivity, creativity, dynamism, and research orientation (Ansari & Khan, 2020; Hidayati & Yuniati, 2021). Studies in a number of disciplines have demonstrated the influence of social media on learning outcomes. For example, in a study in science disciplines, researchers observed the progression to more sophisticated discussion on social media (Ozturk et al., 2021). In another experiment with social media use by future teachers of English as a Foreign Language, the authors found evidence of the improvement of diverse language skills (Pitaloka et al., 2021).

Researchers have previously defined limitations and barriers for technology integration in educational contexts such as hardware scarcity or teachers’ attitudes and beliefs (Ertmer & Ottenbreit-Leftwich, 2013; Prestridge, 2010; Teo, 2009). Teachers’ attitudes and beliefs about social media could, for example, help explain why many students consider social media to be more appropriate for informal social communication than for learning in formal contexts (Karakoyun & Lindberg, 2021). Because of the complexities of using social media, it is important to give PSTs early experiences and guidance with educational social media use so that they can utilize these media wisely as future teachers (Carpenter & Krutka, 2015). For example, recent research has observed the need to carry out social media learning activities in teacher education with a critical approach (McGarr & Gallchóir, 2020).

International and comparative research has addressed the integration of social media in different contexts and has shown that PSTs from different countries may use and perceive social media differently. For example, a previous survey study by Carpenter et al. (2016) found that PSTs from the USA envisioned more future usage of Twitter for educational purposes than Spanish PSTs. Karakoyun and Lindberg (2021) analyzed qualitative data from a survey of 201 PSTs from Turkey and Sweden. The authors found out that PSTs from both countries perceived social media as being mainly for communication and access to information, and only a few of them connected it to education. Also, Turkish PSTs had more negative perceptions of social media—connected to addiction—than the Swedish ones (Karakoyun & Lindberg, 2021). In another comparative study, Prestridge et al. (2021) explored ICT expert teachers’ professional learning through social media in relation to socio-technical affordances, involving 15 teachers from Australia, Belgium, and the USA, and reported that the examination of cultural values toward competition was key to understanding these teachers’ social media use (Prestridge et al., 2021). In another comparative study between Germany, Israel, and Spain, Spanish PSTs perceived a greater impact of social media on learning than German PSTs, while also showing higher sense of identity, control, and ownership of social media (Buchem et al., 2020). International comparative research in teacher education contexts has given only scant attention, however, to PSTs’ perceptions of data privacy matters related to social media use in education contexts.

### Social media data privacy considerations in pre-service teacher education

In the context of social media, two specific types of privacy apply (Livingstone et al., 2019): the interpersonal, which refers to “how a ‘data self’ is created, accessed, and multiplied via social connections,” and the commercial, which relates to “how personal data is harvested and used for business and marketing purposes” (p. 4). Although social media are used globally, how much users share and how they manage their interpersonal privacy are culture specific (Trepte et al., 2017). Commercial privacy may also vary depending on legal frameworks and policies in different countries.

Considering these aspects and following the framework of Ur and Wang (2013), three factors must be taken into account in order to better understand cultural differences around social media data privacy matters in education contexts: legal issues (legal frameworks and policies), cultural norms (cultural values), and user expectations (attitudes and awareness). Although the results of this study directly address user expectations, and indirectly may reflect cultural norms, we present legal issues as well here as helpful context for the reader.

#### Legal frameworks and policies

Legal frameworks for social media data privacy differ around the world. In the case of the European Union, the General Data Protection Regulation (GDPR) is considered “one of the main modern advancements in personal data protection laws anywhere in the world,” and it establishes what is legally required of any company to manage EU citizens’ data (Schwartz, 2019, p. 33). Importantly, the GDPR grants personal data ownership to the individual (Ifenthaler et al., 2016). Arguably, worldwide data privacy policies have positively changed thanks to the 2018 introduction of the GDPR, but there remains room for policy improvements (e.g., clarifying the right to delete one’s own data; Nokhbeh Zaeem & Barber, 2020). In addition, the GDPR assumes that individuals can make informed decisions in interacting with complex online data consent mechanisms from different services, which may not always be the case (Vartiainen et al., 2022). In the German context, federal regulations are strict regarding the adoption of software in education, forbidding teachers to use technologies that are not compliant with EU standards of privacy and data protection (Kerres, 2020). On the other hand, in the USA there are relatively few universal regulations related to social media, and companies have largely been expected to regulate their own data practices, with ownership of collected data belonging to these companies (Ifenthaler & Schumacher, 2016; Schwartz, 2019).

In addition to data regulations, some studies suggest that education policies may also influence social media use by teachers for educational purposes, including teacher professional development (e.g., Twitter in the USA, see Greenhalgh et al., 2021). Supranational policies or guidelines that refer to teachers and the digital, such as digital competence frameworks, also need to be taken into account. Both DigCompEdu, the digital competence framework for educators in Europe (Redecker, 2017), which has been adapted in different European countries (including Germany and Spain), and the International Society for Technology in Education (ISTE; 2019) teacher standards in the USA, list different competencies and standards that educators should develop in relation to digital contexts (see Table 1). In New Zealand (NZ) there was at the time of writing no specific digital competence framework for educators, although a general digital competence framework is defined for any citizen (e-Competencies; Ministry of Education, New Zealand Government, n.d.).

**Table 1:** Correspondence of areas of the European framework for educators and ISTE standards according to similarity

European Framework for the Digital Competence of Educators (DigCompEdu, 6 areas)	Educator Technology Standards in the ISTE (ISTE, 7 areas)
Professional engagement (focus on professional environment)	Learner (improving practice by learning from and with others)
Digital resources (sourcing, creating, and sharing)	Collaborator (with colleagues and students to improve practice and share resources)
Teaching and learning (managing and orchestrating the use of digital tools)	Designer (of authentic, learner-driven activities and environments)
Facilitating learners’ digital competence (DigCom: information and media literacy, digital communication and collaboration, digital content creation, responsible use, and digital problem solving)	Facilitator (of learning with technology to support student achievement of the ISTE standards for students)
Empowering learners (accessibility and inclusion, personalization, actively engaging learners)	Leader (to support student empowerment and success)
Assessment (strategies, analysis of evidence and feedback)	Analyst (understanding and using data to drive instruction and support students)
	Citizen (inspiring students to positively contribute to and responsibly participate in the digital world)

Based on Redecker (2017) and ISTE (2019)

The three digital competence frameworks (DigCompEdu, ISTE, and NZ e-Competencies) associated with the four contexts of this study are connected to matters of data privacy in the use of social media in education contexts. The DigCompEdu framework in the EU includes elements related to security, safety, and ethics through the term “responsible use” (Redecker, 2017). Furthermore, in the recently released DigComp 2.2, the European digital competence framework for citizens (Vuorikari et al., 2022), the development of the competence area “Safety,” and particularly, the competence “Protecting personal data and privacy,” includes specific data privacy considerations for digital environments (e.g., identify privacy policy statements regarding how personal data is used in digital services). In the case of the ISTE teacher standards, the facilitator and citizen areas relate to responsible use and data privacy in education. Finally, considering the NZ e-Competencies areas, these elements could have their place within the e-awareness category, which refers to developing awareness of ICTs and their relevance in society, including digital citizenship. This latter emphasis is in line with the “citizen” area of ISTE.

Taken together, these frameworks and laws underscore the need for teachers themselves to develop digital competencies related to social media use and data privacy matters in education contexts, and for teachers to foster the acquisition of those competencies in younger generations.

#### Cultural values

Teachers’ and PSTs’ social media use, and associated data privacy issues, can be related to contextual norms and backgrounds (e.g., university and schooling context, local context, teacher’s values; see Prestridge et al., 2021; Greenhalgh et al., 2021; Milton et al., 2021), and national cultural values and variables (Hofstede, 2001, 2010; Ronen & Shenkar, 2013). To shed light on differences related to the countries of the PSTs in our sample, in what follows we make cautious use of Hofstede’s (2001, 2010) cultural dimensions to explain some cultural differences in educational contexts, acknowledging the existence of diverse cultural backgrounds beyond Hoftstede’s dimensions.

The dimensions of individualism and uncertainty avoidance have been identified as being especially relevant for Internet use and privacy calculations regarding social media (Trepte et al., 2017), and important differences on these dimensions among the countries in our research have been noted (e.g., Schwartz, 2019). Analysis of these dimensions suggests the USA “has one of the most individualistic cultures in the world … and the tendency of individualistic cultures [is] to devalue information privacy in exchange for higher values on ‘getting ahead’ socially or economically” (Schwartz, 2019, p. 24). Indeed, in research by Prestridge et al. (2021), a competitive culture regarding sharing practices in social media was found among US teachers. Additionally, German individuals typically display higher uncertainty avoidance, as they are “less likely to be open to things that seem riskier than are individuals in the US” (Schwartz, 2019, p. 30). Cultural values regarding individualism and uncertainty avoidance may suggest that PSTs in Germany and Spain would be less likely to use social media than PSTs in the USA and New Zealand. Due to the possible associated risks, German and Spanish PSTs also might be relatively more likely to consider the effects of the choices they make on social media on their social group than their US and New Zealand counterparts.

Furthermore, Hofstede’s (2010) dimensions of long-term orientation and indulgence-restraint may offer additional insights related to Internet use and privacy considerations regarding social media. Cultures with a more long-term orientation, such as Germany, may be less likely to embrace immediate benefits such as connections via social media that come at greater long-term cost such as risks to data privacy. German PSTs could, therefore, be more likely to be reserved and cautious in using social media or demonstrate indulgence-restraint by using restrictive data privacy configurations (Schwartz, 2019). German caution related to the use of digital technologies in education has been highlighted in the literature, possibly linked to German historic experiences of surveillance and skeptical attitudes toward technology (Kerres, 2020).

In addition to Hofstede’s cultural dimensions, the ecocultural framework takes into account the role of religion, language, and geography across countries (Ronen & Shenkar, 2013). According to the cultural clustering of countries developed by Ronen and Shenkar (2013), the sample in this study includes representation of the Latin Europe global cluster (Spain), the Germanic cluster (Germany), and the Anglo cluster (New Zealand and the USA). The latter is characterized by dimensions that connect to Hofstede’s (2010) cultural dimensions: high in individualism, and low in uncertainty avoidance. Ronen and Shenkar (2013) also point out economic variables as potentially important to cultural variation. In this regard, the countries involved in this study are part of the group of clusters with high gross domestic product based on purchasing power parity (GDP-PPP).

#### User attitudes

Social media users can have ambivalent and even contradictory attitudes toward social media use. Research suggests many users may lack knowledge concerning data, algorithms, and online privacy, while at the same time, discomfort with data practices has been noted (Sander, 2020). Inattention to protecting one’s own personal data has also been highlighted (Obar & Oeldorf-Hirsch, 2018).

In the context of teacher education, several studies have addressed aspects of personal data privacy and PST’s beliefs and attitudes. Gallego-Arrufat et al. (2019) identified that nearly half of Spanish and Portuguese PSTs who participated in a survey (*N* = 317) demonstrated insufficient awareness of fundamental concepts related to personal data privacy such as digital identity, footprint, and online reputation. A study with 176 Spanish PSTs based on the DigCompEdu framework showed that although PSTs had relatively high levels of digital competence, those levels did not progress over time, which could mean that digital competence was not addressed during their teacher education (García-Vandewalle et al., 2021). In particular, the authors concluded that the main limitation in the Spanish PSTs’ digital competence is their lack of skills for digital security (competence area “Safety” in the DigComp framework).

Similarly, a survey study, focused on copyright and privacy in social media carried out with PSTs in Malta, Norway, and Spain (*N* = 1,131), found that, overall, participants from the three countries showed a lack of awareness of responsible behavior and of knowledge in terms of copyright and privacy in social media, but with significant differences in awareness between countries (Milton et al., 2021). The authors of this study recommend that all teacher education programs should include copyright and privacy in social media contexts in their curricula.

An interview study with Finnish PSTs (*N* = 14) revealed that participants shared similar strategies to protect their digital identities in social media environments (e.g., adjusting profile privacy settings), but experienced difficulties in enacting data agency due to their lack of knowledge concerning commercial-level data issues (Vartiainen et al., 2022). The authors argued that, without a sophisticated understanding of such issues, “it is unlikely that these future teachers would be prepared to facilitate children and youth’s agentive actions in data-driven society” (p. 15).

Investigating PSTs’ perceptions and expectations is important because PSTs’ beliefs and intentions related to technology inform their actions (McGarr & Gallchóir, 2020; Nelson & Hawk, 2020), and are key in order to consider the impact of technology on students’ learning and performance (Ertmer & Ottenbreit-Leftwich, 2013; Tondeur et al., 2019). In addition, even though some research considers cross-cultural comparisons (Milton et al., 2021), cultural differences are still under-addressed in the literature regarding social media and data privacy issues in education contexts, despite likely playing a key role in such settings.

Building on what is known about PSTs’ beliefs and practices in comparative studies, this work, therefore, addresses a gap in the literature by adding to the research on cross-cultural differences in PSTs’ perceptions concerning the use of social media in education, their attitudes and beliefs regarding data privacy, and the implications for their future teaching practices. Furthermore, this study can be related to national and international educational frameworks for teachers’ digital competence in which student empowerment is an important area.

## Materials and methods

### Instrument

This study utilized an anonymous online survey that included closed-ended items in combination with optional open-ended prompts to explain responses to the closed items. In line with our research questions, the 50-item survey identified PSTs’ beliefs and attitudes regarding educational and professional social media uses and related matters of data privacy. The closed-ended items were primarily Likert scale items that used a five-point scale. The authors from Germany, Spain, and the USA collaboratively designed the survey, which was informed by their prior work (Carpenter et al., 2016; Tur et al., 2017; Zawacki-Richter et al., 2019), as well as other relevant literature on professional and education uses of social media (Smith Risser, 2013; Xing & Gao, 2018). A draft of the survey went through expert review (Olson, 2010) by researchers from both Spain and the USA, and edits were made based on feedback from four reviewers. For example, one reviewer noted that several of our draft items were double-barreled questions, and we therefore split them into separate items. Another reviewer suggested that we provide examples of the “problems associated with social media” referred to in one survey item, and we accordingly added a parenthetical comment: “(e.g., cyberbullying, spamming, phishing).” Once the survey was finalized in English, translations into Spanish and German were drafted and checked by native Spanish and German speakers. Minor edits were made based on their feedback. The survey was first used in a previous work by the Marín et al. (2021).

Following an item related to providing informed and voluntary consent, the survey featured items divided into five parts that collected data on: (1) participants’ demographic characteristics, (2) their knowledge of use of social media for professional development (PD) by in-service teachers, and their own expectations for social media use, (3) their awareness concerning social media use for educational purposes by in-service teachers, and their beliefs about who bears responsibility for teaching about social media, (4) their knowledge of social media companies’ data privacy policies, and (5) their degree of comfort concerning social media companies’ data privacy policies. The questionnaire used is included in Online Appendix 1.

### Context

The participants in this research were PSTs at one university in each of Germany, New Zealand, Spain, and the USA. While the US university was private, the others were public. All of the universities were small to medium size in terms of number of students (between 7,000 and 16,000) and were serving mainly national or regional student populations, rather than international ones. In addition, in the four universities, teacher education occupied important positions within their academic offerings. Teacher education programs vary depending on the country, as regulations for the teaching profession differ across nations. Therefore, the following paragraphs provide additional information about the populations of students from which the sample was drawn at each university. Also, relevant institutional policies regarding social media use and/or data privacy and security in place at each university at the time of the research are mentioned.

German participants were in various years of their Bachelor level studies, and were studying two different disciplines (e.g., Biology, English), and following curricula for teaching at different school levels (primary, secondary or vocational education). The pedagogical curriculum at the German university included digital technologies as part of elective courses. The total number of students studying the pedagogical coursework—a set of courses—for teacher education within their double degree was around 900 students^1^ at the time of the study. At the time of the research, no institutional policies regarding the use of social media were identified in teacher education, but there were guidelines on general aspects of social media use at the institutional level, which included a call for attention to platforms’ terms of use. The university’s data privacy and security guidelines adhered to the GDPR.

In New Zealand, participants were at the graduate level in a one-year initial teacher education program for teaching in the primary school sector. In this program, the use of digital technologies was taught as part of required curriculum content courses, rather than in a separate, dedicated education technology course. In total, 114 students were part of this teacher education program at the time of the study. Policies regarding the use of social media that were specific to teacher education did not exist at the time of the study, but a social media policy existed in order to regulate its broader institutional use. The data privacy policy of the university explained how it collects, uses, and shares personal information from its community, and covered issues of consent, access to information by the individuals, staff access to confidential information, security and retention of information, privacy impact assessments, privacy complaints, and the student agreement.

Spanish participants were taking either a 4-year undergraduate program for Childhood and Primary Education or an MA program for Secondary Education teachers. In both cases, digital technologies had a relatively limited presence, with one or two compulsory courses for the former and none required for the Secondary Education participants. There were 80 total students in these programs at the campus of the university that was involved when this research occurred. When the study was conducted, no specific institutional policies regarding the use of social media were in place in teacher education; only general guidelines for the broader institutional use of social media existed. The university’s data privacy and security guidelines adhered to the GDPR.

Participants from the USA were preparing to become primary or secondary school teachers through their studies in one of ten different undergraduate programs. These programs all included a required one-semester educational technology course which featured an introduction to professional and educational uses of social media. Additionally, educational technology was also meant to be addressed in pedagogy and assessment courses. There were 212 undergraduate teacher education students at the university when the study was conducted. In terms of institutional policies regarding the use of social media, the handbook of regulations for PSTs’ student teaching experience mandated that PSTs “are prohibited from interacting with public school students through social media such as Facebook or Twitter.” Additionally, the university’s data privacy policy specified how and what data from its community it collects, and the data uses, and it stated that the university will not sell personally identifiable information to third parties.

### Sample

The sample of participants was of convenience. Two-hundred twenty-five individuals completed the questionnaire prior to the pandemic. 32% (*n* = 71) came from the German university, 32% (*n* = 71) were from the US university, 25% (*n* = 57) were from the Spanish university, and 12% (*n* = 26) were from the New Zealand university.

Regarding gender, 78% (*n* = 176) identified as female and 22% (*n* = 49) as male. These numbers correspond with females’ overrepresentation in teacher education programs in the four countries. The mean age was 24.0 years old (SD = 6.6). Regarding the educational levels the participants were preparing to teach at, the most common ones were secondary education (49%) and elementary or primary education (40%).

### Data analysis

Quantitative data were analyzed using SPSS Version 26 and based upon guidance in resources from Laerd Statistics (2015). First, descriptive statistics were produced. Then, to investigate differences between the groups of participants from the different universities on Likert scale items, we used a between-subjects design with the universities as the independent variable, and the participants’ attitudes or perceptions as the dependent variable. Because of the ordinal nature of the Likert scale items, non-parametric approaches were used to analyze the data. We ran Kruskal–Wallis H tests (Kruskal & Wallis, 1952) to determine if there were statistically significant differences between the groups of participants from the different universities on various ordinal dependent variables. For Kruskal Wallis H tests, each group should have a size of 5 or more (Laerd Statistics, 2015), and in our data, each group had an *n* = of at least 26. We ran chi square tests of homogeneity to investigate group differences on two dichotomous survey items.

The data from optional open-ended items were meant to supplement and help explain the quantitative data. Sixty total responses were given across these four items; when appropriate, responses were translated into English. Two of the authors reviewed and discussed the responses to identify data that provided details or examples that could contribute to the presentation of the survey results. In order to organize these data, the responses were deductively grouped according to the topics in bold presented in Sect. *Results and Discussion* (Saldaña, 2021), although not all topics had qualitative data to refer to.

### Limitations

As stated previously, our sample was one of convenience and was limited to one university in each of the countries involved in the study, and included participants from different types of pre-service teacher populations according to each country’s regulations for the teaching profession. These institutions do not represent the entirety of higher education in each of the countries, nor can the participants be taken to represent all PSTs from the countries where the universities are located. Also, our study relies heavily on quantitative analysis, which could make it difficult to interpret the nuances in the results, and our main data source is self-reports from the same PSTs. It should be noted that the instrument was primarily validated by expert review, and future work could consider additional means for validation of the survey. Finally, differences across countries in terms of social media data privacy regulations, perceptions, and awareness of social media data privacy issues, as well as use of digital competence frameworks, mean that the participants may have had different understandings of what some of the survey items meant. Our findings should be considered bearing in mind these caveats.

## Results and discussion

### Use of social media across the sample

**Fig. 1:**
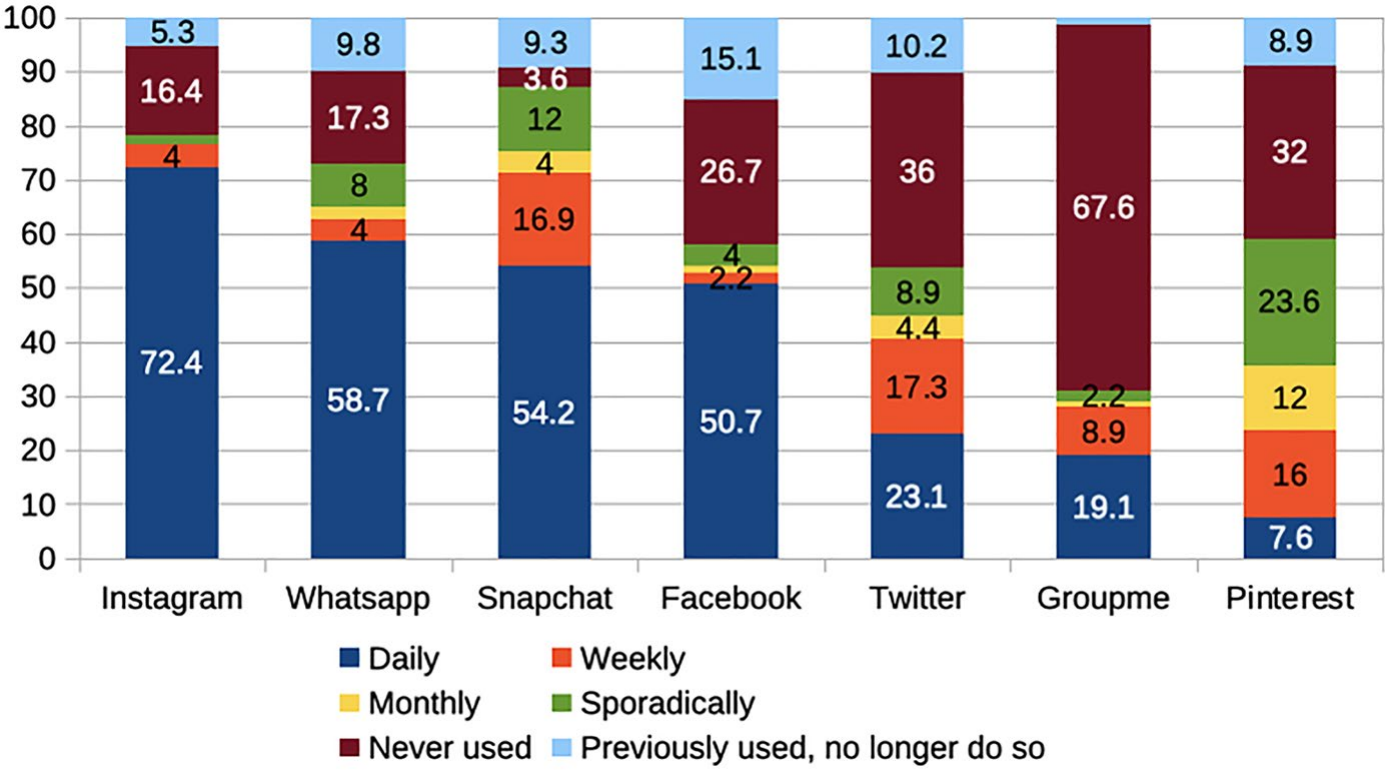
“How often do you login to the following social media?” (%)

To contextualize our findings related to our research questions, we first provide information regarding participants' self-reported frequency of use of various social media platforms or apps. Many participants were frequent users of social media (see Fig. 1); 99.1% of PSTs indicated at least daily access to one social media service, and 85.4% indicated they accessed two or more services daily.

The participants from the various universities used many social media platforms at comparable rates. However, WhatsApp was used by all the German and Spanish participants but only half of the New Zealand participants, and one-third of the US participants. The low usage of WhatsApp in the USA can be explained by looking at the development of telecommunications in the country. US citizens had cheaper texting services than other countries, did not need much international texting due to low rates of traveling abroad, and already used other Messenger apps (e.g., Facebook Messenger) at the time in which WhatsApp appeared, so they did not need another app for the same purpose (Shwayder, 2019). Another noticeable difference was that GroupMe, while used by all but one of the US participants, was not used by any of the German, Spanish, or New Zealand participants.

The subsections that follow address in turn each of our research questions.

### RQ1. What are pre-service teachers’ beliefs about educational and professional use of social media?

**Fig. 2:**
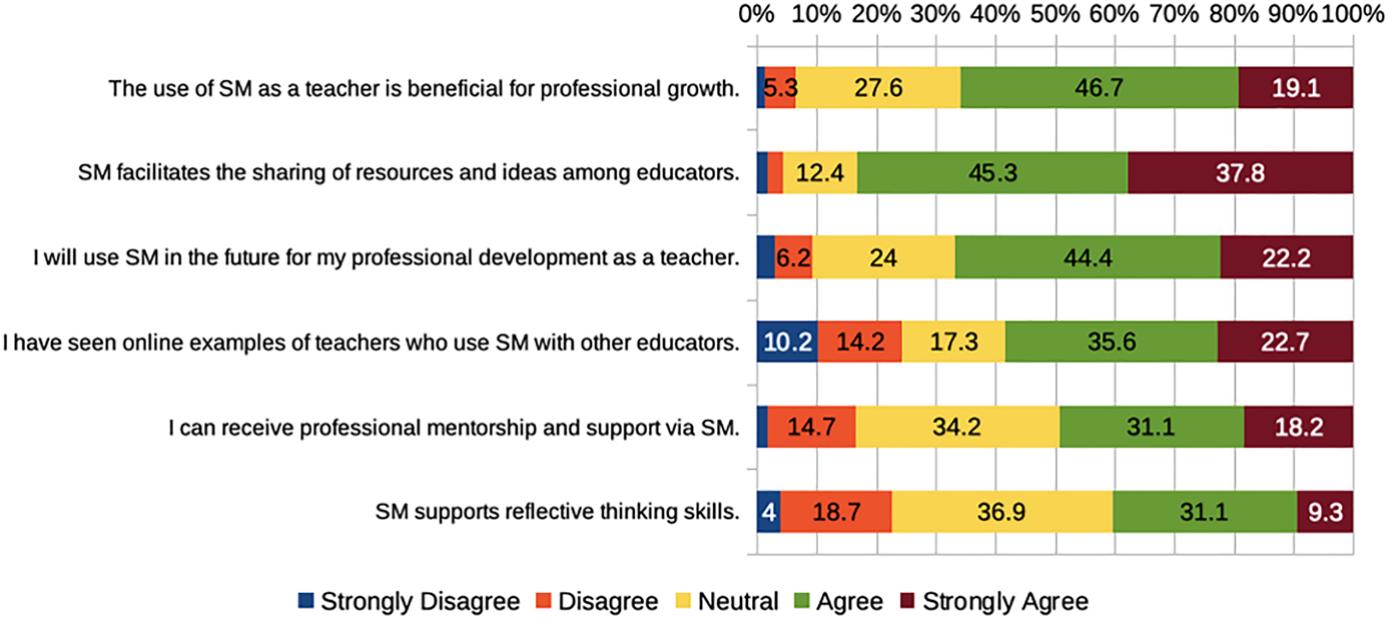
PTSs’ beliefs about professional use of social media. *SM* = social media

Overall, many of the participants appeared to see potential value in professional use of social media, as seen in previous research (Carpenter & Krutka, 2015; Carpenter et al., 2016; Prestridge et al., 2021). Almost two-thirds of PSTs (66%) agreed or strongly agreed that social media can be beneficial for professional growth, and exactly two-thirds anticipated future use of social media for professional development (PD; Fig. 2). In terms of specific aspects of PD, participants were much more likely to agree or strongly agree with the statement that social media “facilitates the sharing of resources and ideas among educators” (83%) than that social media can help them “receive professional mentorship and support” (49%) or that social media can “support reflective thinking skills” (40%) among teachers. This reflects findings from Prestridge et al. (2021), where teachers tended to interact with artifacts (e.g., resources) more than engaging in networking (e.g., through professional mentorship).

While some participants noted positive aspects around using social media for PD, others stated concerns about teachers using social media. For example, one wrote that “the one thing I worry about with educators on social media is the constant comparing of each other and the question, ‘If I am not creating social-media-worthy lessons, am I not as good of a teacher?’” These kinds of concerns were also identified among teachers in Prestridge et al. (2021), where one of the categories that underpinned teacher reasoning and action through social media was competition.

**Fig. 3:**
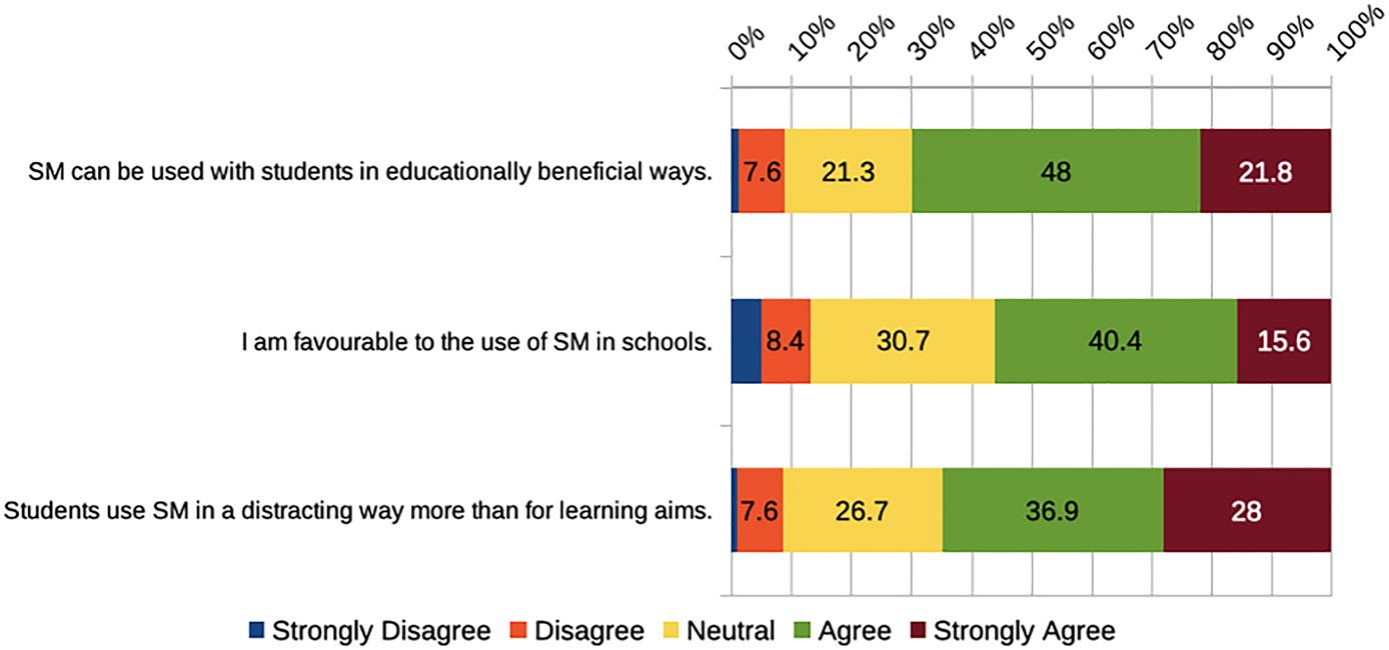
PSTs’ beliefs about the educational use of social media

More than two-thirds of the participants (70%) agreed or strongly agreed that social media can be utilized with students in ways that benefit education. Just over half (56%) reported being favorable to social media use in school settings (Fig. 3), in line with previous studies by Carpenter et al. (2016) and Tur et al. (2017) where students could envision the impact of their current learning in their future teaching. However, these findings contrast the results in Karakoyun and Lindberg (2021), where only a few PSTs connected social media to education.

Some respondents had different views of using social media in the classroom as can be seen in this quote, “Social media is tricky because it can be a huge distraction as well as a place for bullying and hate.” Almost two-thirds of respondents (65%) agreed or strongly agreed that students tend to employ social media more as a distractor than as a tool to learn (Fig. 3). This finding can be related to negative views from PSTs regarding social media in Karakoyun and Lindberg’s (2021) research.

**Fig. 4:**
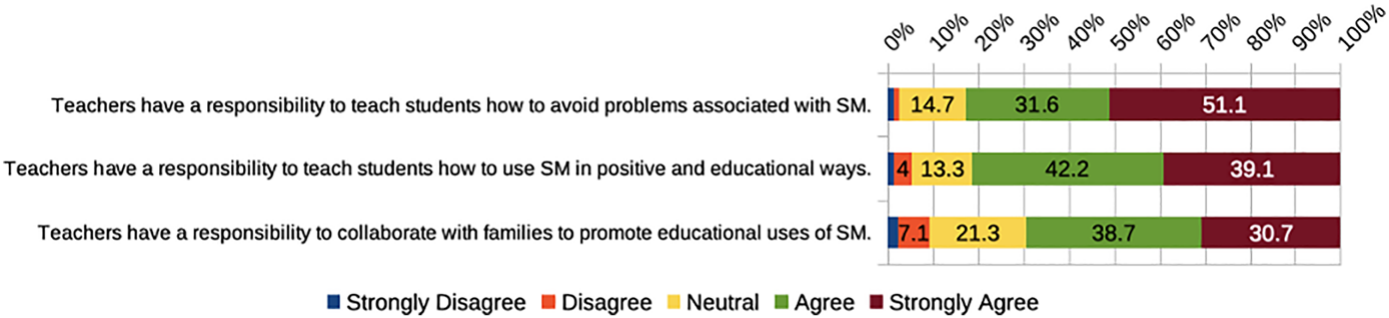
PSTs’ beliefs about teachers’ responsibility concerning the use of social media

Participants were overwhelmingly willing to accept responsibility for teaching students about social media (Fig. 4). More than 80% embraced the related statements (teachers have a responsibility to teach students how to avoid problems associated with social media, and how to use social media in positive and educational ways). These findings align with research by Vartiainen et al. (2022), where PSTs saw themselves as responsible actors in educating future generations in digital environments. Also, in line with digital competence frameworks in place, this role is part of the competences that are expected from teachers. In the case of the DigCompEdu, the competence areas “Facilitating learners’ digital competence” and “Empowering learners” have special emphasis on this implication (Redecker, 2017). Similarly, in the case of the ISTE (2019) standards for educators, the areas of “Facilitator,” “Leader,” and “Citizen” involve this supportive role. In NZ e-Competencies areas (Ministry of Education, New Zealand Government, n.d.), the e-awareness category fits this purpose, as well. One participant elaborated on factors associated with students using social media:

Technology has the power of transforming a student’s learning experience, if used right. Students need to be educated on how to use it [social media] in an educational way so that their overall learning experience can be improved. By using social media, students can gain knowledge not just from their teacher, but from other professionals and students.

This quote exemplifies PST willingness to take responsibility for educating students on educational uses of social media.

**Fig. 5:**
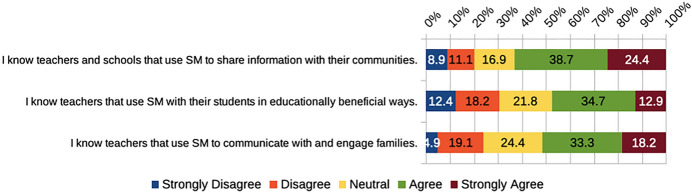
PSTs’ beliefs about role models related to social media

PSTs’ belief in the educational potential of social media may be connected to the fact that many of them seemed to have at least some role models of educator social media use (Fig. 5). Such role models could have been from PSTs’ time as primary or secondary students, or could potentially result from their teacher education experiences, if educational use of social media was modeled therein (Carpenter & Morrison, 2018).

### RQ2. Are there differences in the perceptions of PSTs from universities in four countries concerning the educational and professional use of social media?

Nineteen of the survey’s Likert scale items pertained to educational and professional use of social media. The results of the Kruskal–Wallis H tests to determine if differences existed across university groups can be found in Online Appendix 1. For three of the 19 items, there were no statistically significant differences in levels of agreement across the university groups, *p* > 0.05; in other words, the distribution of responses from the groups of students from the different universities were similar. For the remaining 16 items, there were statistically significant differences, *p* < 0.05. In 13 of these 16 cases, pairwise comparisons using Dunn's (1964) procedure with a Bonferroni correction for multiple comparisons identified one or more cases in which pairs of university groups were significantly different.

USA PSTs were most likely to agree or strongly agree with almost all of the items related to professional uses of social media, while German (GER) PSTs were the least likely to agree or strongly agree. For example, for the item, “I can receive professional mentorship and support via social media,” there was a significant difference in the level of agreement across the university groups. Subsequently, pairwise comparisons were performed using Dunn's (1964) procedure with a Bonferroni correction for multiple comparisons. This post hoc analysis revealed statistically significant differences in agreement levels between USA PSTs (*Mdn* = 4) and GER PSTs (*Mdn* = 3), but not between any other group comparison.

On three items related to teachers’ responsibility to teach about social media, the New Zealand (NZL) participants were the most likely to be in agreement, especially in comparison to USA participants. So, while USA PSTs seemed in many ways to be the most positive regarding educational uses of social media, they were not as willing to embrace responsibility for teaching about social media. Despite differences in their perceptions of various specific aspects of the use of social media for PD, for the general statements (use of social media in the future for PD as a teacher and favorability to its use in schools), there were not significant differences between the groups from the four universities.

Overall, the USA PSTs in our study seemed to be more in agreement with statements regarding educational uses of social media than the other groups of PSTs, and this was particularly the case regarding survey items related to professional uses of social media. This result could be interpreted through the dimension of individualism and uncertainty avoidance (Hofstede, 2001, 2010) and the consideration of the Anglo cluster (Ronen & Shenkar, 2013). The higher value on individualism in the USA could be associated with PSTs advancing themselves forwards professionally through their social media use, and a lower value in uncertainty avoidance could contribute toward USA PSTs being more open to social media and its associated risks than the other groups (Schwartz, 2019). Also, findings regarding professional individualism would be aligned to the competitive category emerging from the data analysis of USA teachers’ interviews in Prestridge et al. (2021). It should also be noted that many of the USA PSTs had taken a course which, among various topics, included a specific introduction to professional and educational uses of social media,

In seven cases, which were mostly connected to teachers’ responsibility to teach about social media, NZL PSTs were statistically significantly more likely to agree with statements than one of the other groups of PSTs. These results would not be explained, for example, by a high value for individualism nor by the characteristics of the Anglo Cluster (Ronen & Shenkar, 2013), and may be partially explained through other means (e.g., the conception of digital citizenship within NZ e-Competencies, or characteristics of the teacher education program regarding the incorporation of digital technologies). This sense of teachers’ responsibility was also highlighted by Finnish PSTs in the study by Vartiainen et al. (2022). On only one occasion were GER PSTs significantly more likely to agree with statements than one of the other groups of PSTs. This may be interpreted through a higher value to long-term orientation in GER PSTs, who could be more reserved and cautious in using social media than other groups, but also, potentially, to strict German federal regulations regarding the use of social media tools in education and generally more skeptical attitudes toward digital technology in education in Germany (Kerres, 2020).

### RQ3. What are pre-service teachers’ beliefs about and awareness concerning data privacy matters in social media services in educational contexts?

**Fig. 6:**
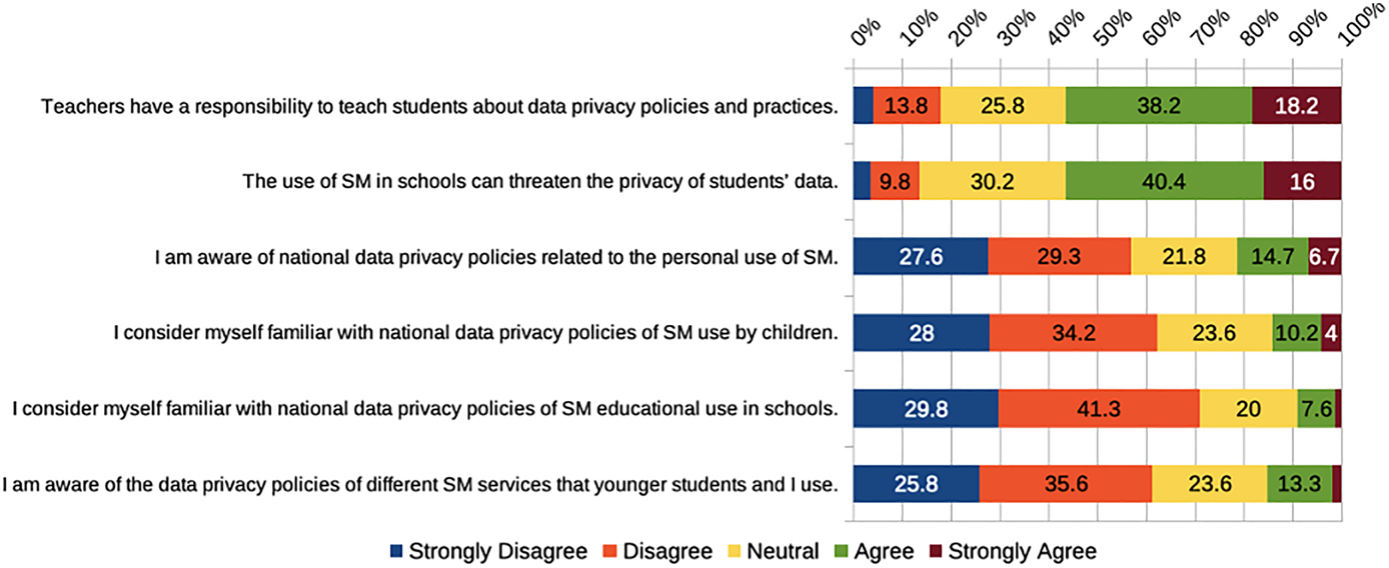
PSTs’ beliefs regarding data privacy policies and social media practices

Approximately 56% of the PSTs agreed with both the statements that “Teachers have a responsibility to teach students about data privacy policies and practices,” and that “The use of social media in schools can threaten the privacy of students’ data” (Fig. 6). The idea that teachers are responsible for teaching students about data privacy practices is illustrated in quotes from two of the participants, “I think that teachers are responsible for dealing with problems and for behavior when they [students] use them [social media],” and “It is important that teachers are able to teach students about the harms that social media can do.”

Despite this apparent support of the importance of teaching about data privacy and awareness of potential privacy problems associated with the use of social media, some *challenges regarding data policies* were identified. For instance, 72% of participants reported that they had never read a privacy policy of a social media service. Similarly, only 15% of the participants agreed or strongly agreed that they were cognizant of the data privacy policies of widely used social media platforms (Fig. 6). Almost half of the participants (48%) were uncertain whether the data privacy policies in their countries permitted them to employ social media for professional purposes and more than half (56%) were uncertain whether those policies allowed them to use social media for educational purposes with school students. Only 21% indicated they were acquainted with national data privacy policies regarding personal social media use, and even fewer (14%) considered themselves informed of those policies connected with children’s utilization of social media. Despite the aforementioned majority support for the use of social media in schools, just 9% of participants felt they were apprised of those policies concerning the educational use of social media with students in educational settings (Fig. 6).

On the whole, many of our participants’ beliefs coincide with the findings of previous research that showed a dearth of knowledge pertaining to data privacy in general populations (Obar & Oeldorf-Hirsch, 2018; Sander, 2020), and among PSTs (García-Vandewalle et al., 2021; Milton et al., 2021). Our findings align with research that has highlighted an absence of teacher preparation for issues related to personal data awareness and practices (Gallego-Arrufat et al., 2019), especially related to commercial privacy (Vartiainen et al., 2022). Beliefs associated with the potential of social media in education could be connected to having educator role models of technology use (see Nelson & Hawk, 2020). Logically, teacher educators are among those who could serve as such role models, and thus support data literacy development in teacher education programs (García-Vandewalle et al., 2021; Milton et al., 2021). Teacher education programs could be an example for PSTs of a possible suitable balance of data control, use, and sharing between students and educational institutions (Ivanova et al., 2019). PSTs themselves need to develop personal data literacy skills, and the pedagogical strategies to address associated issues in schools. These skills are essential elements of digital competence (Arreguit O’Neill, 2019). Educators must be able to recognize related risks, critically reflect upon data literacy practices, assess new developments, and make data-informed decisions (Sander, 2020), which calls for the development of data agency (Vartiainen et al., 2022). Digital competence frameworks for educators could also incorporate these elements.

**Fig. 7:**
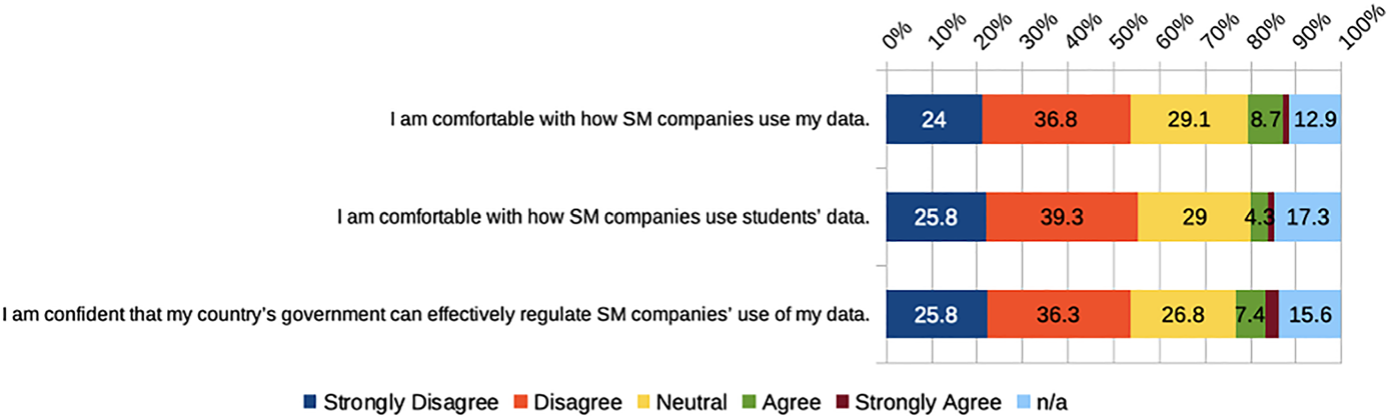
PSTs’ level of comfort and confidence regarding social media companies’ data privacy practices

Participants tended to be uncomfortable with what social media companies do with their data (61%) and student data (65%) (Fig. 7). For example, one participant elaborated, “I wish social media companies were more clear about what they use the data for.” In addition, most PSTs (62%) did not think that their countries’ governments were able to successfully regulate what use these companies make of their users’ data.

These findings reflect insights related to data agency at the commercial level and could be linked to complicated legal language or non-participation in social media being perceived as an unrealistic option (Vartiainen et al., 2022). The complexity of privacy concerns is reflected through human contradictions or dilemmas between concern, convenience, and resignation (Sander, 2020).

Also, findings here bring additional insights into how PSTs perceive governments’ regulation of social media companies, which may be related to national, regional, and local policies in force (Greenhalgh et al., 2021).

### RQ4. Are there differences between PSTs from universities in the four countries’ beliefs about and awareness concerning data privacy in social media?

In terms of awareness, there was a statistically significant difference between the groups in terms of the proportion of students who had read a privacy policy of a social media tool they used, χ^2^(3) = 8.731, *p* = 0.033. SPA (Spanish) participants were the most likely to have read a privacy policy, followed by GER, then NZL, then USA. Post hoc analysis involved pairwise comparisons using the z-test of two proportions with a Bonferroni correction. The proportion of SPA students who had read privacy policies was statistically different from the proportion of USA students, *p* < 0.05.

Fifteen of the Likert scale items pertained to data privacy matters. For these items, Kruskal–Wallis H tests were run to determine if there were differences in degree of agreement or disagreement with the statements included in the Likert scale items between the groups of students from the four universities. The results of these tests can be found in Online Appendix 2. For four of the 15 items, there were no significant differences, *p* > 0.05; in other words, the distribution of responses from the groups of students from the different universities were similar. In the remaining 11 tests, there were statistically significant differences, *p* < 0.05; i.e., the distribution of responses from the groups of students from the four universities were different. In eight of these 11 cases, pairwise comparisons using Dunn's (1964) procedure with a Bonferroni correction for multiple comparisons identified one or more cases in which pairs of university groups were significantly different.

For the items regarding comfort with how social media companies use educator and student data, there were significant differences in the level of agreement across the university groups. For the item about their own data, post hoc analysis revealed significant differences in agreement levels between USA PSTs (*Mdn* = 3) and GER PSTs (*Mdn* = 2) but not between any other group comparison. For the item about students’ data, post hoc analysis revealed statistically significant differences in agreement levels between USA PSTs (*Mdn* = 2) and NZL PSTs (*Mdn* = 1), but not between any other group comparison.

For the item, “Teachers have a responsibility to teach students about data privacy policies and practices” there was a significant difference in the level of agreement across the university groups. Post hoc analysis revealed statistically significant differences in agreement levels between NZL PSTs (*Mdn* = 4) and USA PSTs (*Mdn* = 3), and between NZL PSTs (*Mdn* = 4) and GER PSTs (*Mdn* = 3), but not between other group comparisons. Like findings from RQ2, NZL PSTs were again the most willing to accept responsibility to teach students about a technology-related topic.

There were no statistically significant differences among the groups from the four universities on three items related to awareness of national data privacy policies, and one item pertaining to the data privacy policies of social media services. In other words, participants from all four universities tended to indicate they were unaware of such policies. However, the distribution of responses from the groups of students from the four universities was statistically different for the item, “I consider myself familiar with national data privacy policies related to the use of social media with educational purposes in the schools.” In this case, SPA participants (mean rank = 2.40) were significantly more likely to agree with the statement than USA participants (mean rank = 1.97). In addition, there was not a significant difference among the university groups on the item, “The use of social media in schools can threaten the privacy of students’ data.”

We noted that the likelihood that the PST groups had read a privacy policy aligned with the levels of uncertainty avoidance in their respective countries (Hofstede, 2001, 2010). SPA PSTs come from the country with the highest uncertainty avoidance and were most likely to have read a privacy policy, with GER, NZL, then USA falling in the same order in the ranking of these two items. Participants from Spain demonstrated higher levels of self-reported awareness with national data privacy policies that concern the educational use of social media. However, a general unawareness regarding data privacy in social media was found in our sample, as has been also identified in other international comparative studies (Milton et al., 2021).

### Implications for policy and practice

Given instances of carelessness with students’ personal data (e.g., Rosenberg et al., 2022) and teachers losing their jobs because of social media missteps (Warnick et al., 2016) more education-specific policies that guide and protect students, PSTs, and teachers may be needed or at least beneficial. The pace of digital technology development can challenge policymakers to formulate regulations and guidance (e.g., Saldaña et al., 2021), but social media’s ubiquity in modern life and education means that schools and teacher education programs need policies that help education stakeholders benefit from the affordances of social media and mitigate the potential challenges and pitfalls.

Addressing data privacy issues in teacher education seems appropriate at the outset of a new decade in which it is probable that the datafication of learning will become mainstream (Selwyn et al., 2020). Furthermore, the recent impact of the COVID-19 pandemic on digital education has been dramatic, and it remains to be seen what the extent of its effects will be in terms of experimentation with large quantities of student data (Williamson et al., 2020).

Our work on PSTs' perceptions of social media and data privacy in education has implications for those involved in teacher education and development. First, digital competence frameworks for educators should more comprehensively address data privacy issues (Raffaghelli & Stewart, 2020), and in doing so could help guide the design or redesign of teacher education programs. This may be useful in order to leverage the awareness regarding social media policies that affect their practice and increase their comfort when responsibly using and teaching about social media, in relation to the beliefs and attitudes that PST in our sample expressed. In the case of the European framework for the digital competence of Educators, DigCompEdu (Redecker, 2017), some such revisions have been proposed; for example, using the expressions “facilitating learners’ data literacy,” and “learners’ responsible use of data” (Raffaghelli, 2019). The adjustment of the recent more general DigComp framework seems to bring some enhancements for the DigCompEdu in this respect (Vuorikari et al., 2022), with specific considerations of data privacy for digital environments.

Secondly, attitudes and beliefs have been observed as key variables for technology integration in school contexts (Scherer et al., 2018), so PSTs would benefit from scaffolded early experiences in teacher education that could influence and inform their attitudes, beliefs, and actions related to social media and data privacy. Neither completely rejecting social media use for learning nor embracing them but ignoring related privacy issues seem to be tenable responses to the presence of social media in education (Marín et al., 2021). PSTs must be knowledgeable about social media platforms if they are to make informed decisions about how and when to use them (Esteve Mon et al., 2018). Arguably, data literacy that includes these issues therefore should be built into pre-service teacher training experiences (Mandinach & Jimerson, 2016). Both the role models in teacher education and the attitudes toward social media in education are positively reflected in our results, but there is still room for improvement for a critical and informed stance, as the findings in the other statements suggest. Furthermore, extending the framework for technology integration barriers in school contexts (Teo, 2009) to PSTs’ attitudes and beliefs concerning data privacy issues in social media could derive new considerations for practice. In addition, understanding algorithmic governance and commercial use of data is a first step so that aspiring teachers can facilitate data agency for future generations (Vartiainen et al., 2022) so that they may feel more knowledgeable and empowered in the use of their data and students’ data than what is seen in our findings.

Therefore, further consideration is required regarding the role of teacher educators as role models and guides for critical reflection and ethical approaches in datafied systems (Zawacki-Richter et al., 2019). Teachers need to deal critically with data literacies, including responsible behavior, copyright, and privacy issues in social media (Marín et al., 2021; Milton et al., 2021; Vartiainen et al., 2022). A critical approach is needed to empower users (in our case, PSTs) to control their own data (Pangrazio & Selwyn, 2019), in line with the underlying assumption of the GDPR. PSTs should also question unethical and non-transparent practices by social media business –such as those that align with neoliberal policies that facilitate control and surveillance (Kühn, 2019; Perrotta & Williamson, 2018; Williamson, 2017) -hence, the importance of understanding social media platforms’ terms of use, in contrast to what many of our participants admitted regarding not reading such terms of use. For example, educators can design personal data literacy activities that include social media data and allow for practicing data identification, data understandings, data reflexivity, and data tactics (Pangrazio & Selwyn, 2019). Practices of this nature, which challenge innovation-centric discourses around educational technology (McGarr & Gallchóir, 2020), suggest broad possibilities for future development of teacher education practices related to social media and data privacy.

## Conclusion

Research on privacy issues, and related data literacies, is still in its infancy and this study contributes to previous work in diverse ways. First, our comparative study includes four national contexts and considers their similarities and differences using an umbrella framework in terms of social media data privacy considerations (Ur & Wang, 2013) that integrates others: legal frameworks and supranational policies—digital competence frameworks—as well as cultural values (Hofstede, 2001, 2010; Ronen & Shenkar, 2013) in the different contexts. Secondly, our research addresses the extent to which future teachers’ digital skills are considered for students’ empowerment, since participants were asked not only about their individual practices but also about their future teaching. Finally, the quantitative approach allows us to contribute with a quite wide and diverse group of participants, which adds insights to those qualitative studies present in the extant literature. Our findings appear to support the idea that as with many other social (and international) phenomena, social media use for educational and professional purposes, and data privacy practices and awareness, vary based on culture and context (Prestridge et al., 2021; Trepte et al., 2017).

Our work makes a valuable contribution to the field through international comparative analysis of PSTs’ optimism and concerns about educational and professional social media use, and awareness regarding data privacy and practices in social media. However, much remains to be learned in terms of using social media and considering associated data privacy issues (Carpenter & Harvey, 2020; Manca, 2020). Future research may consider other elements of the personal data literacies framework from Pangrazio and Selwyn (2019) that go beyond data uses and understandings in social media, for instance, data identification, data reflexivity, and data tactics. Also, investigation is needed of what being *critically data literate* (Sander, 2020) in pre-service teacher training would involve. Three additional research and practice strands may provide insights into how to embed those elements of personal and critical data literacies in existing digital competence frameworks for educators and inform teacher training programs. First, it may be worth exploring the degree of agency that PSTs can exercise and want to exercise over personal data and student data in social media (Selwyn & Pangrazio, 2018). Second, the balance of personal and institutional data control, use, and sharing in formal learning contexts could be investigated (Ivanova et al., 2019). Third, specific social media services on the rise (e.g., TikTok), as well as other social media services designed to mitigate ethics and privacy issues (e.g., Pixelfed), could be explored to examine concrete data privacy practices in the context of pre-service teacher training and in schools. Finally, another area of future research is the analysis and comparison of social media data privacy policies to address key strengths and weaknesses that can inform best practices.

## Supplementary Information

Below is the link to the electronic supplementary material.

Supplementary file1 (DOCX 23 kb)

## Footnotes

1 It is not possible to know the exact numbers of past courseworks.
