# Supplementary material for: Social media and data privacy in education: an international comparative study of perceptions among pre-service teachers
Source: J Comput Educ. 2022 Sep 28:1–27. Online ahead of print. doi: 10.1007/s40692-022-00243-x (PMC9515482; doi:10.1007/s40692-022-00243-x)
Supplement: Supplementary file 1 — Supplementary file1 (DOCX 23 kb) [file 40692_2022_243_MOESM1_ESM.docx]

**Appendix 1 - Questionnaire**

**Survey on Student Teachers’ Perceptions on Social Media**

Welcome to our study on student teachers’ perceptions on social media! We thank you for your interest in this study.

This survey is directed at evaluating your perceptions on the use of social media for educational and professional purposes. With the term social media, we are referring to web tools designed as social networks with the main objective of social communication, such as Facebook, Twitter, Instagram, Snapchat and Whatsapp, but we also consider other social networks designed for learning purposes. This is part of an international study in which we aim to identify beliefs and attitudes of student teachers regarding the educational use of social media and propose educational strategies to support pre-service and in-service teachers in dealing with social media in class.

The approximate duration to answer the survey is between 7 and 12 minutes.

**Development of the study**

Your task is to fill out a questionnaire. You should express your opinion in different ways. There are no correct or wrong answers. For each statement circle the option that corresponds to your opinion.

The type of personal data collected are the age, gender, year of studies and type of school for which you are studying, and they are not connected with your name.

If you still have questions, please contact the researcher in charge of the study at your university.

**Voluntariness and anonymity**

The participation in this study is voluntary. You can discontinue your participation in this study anytime and without giving reasons, and that would not imply any penalty for you.

The data and personal information collected through this study are treated confidentially. For example, those researchers who have personal data through direct contact with you are subject to secrecy. Furthermore, the publication of the results of the study will be done anonymously, which means without that your data can identify your person.

**Data protection**

The collection and processing of your personal data described above is done anonymously in the online questionnaire using a number and without stating your name. The questionnaire survey is conducted using the online survey tool "LimeSurvey". This tool makes it possible to disable the storage of IP addresses and the setting of cookies, so that no conclusions can be drawn on persons. The tool is configured in this survey so that the functions IP address storage and cookies setting are disabled. The IP addresses are thus not saved and no cookies are set. The anonymous data will be stored for at least 10 years.

**Section 0**

**Consent**

(Checkbox) I agree

I have been informed verbally and in writing about the study and its development. I agree to participate anonymously in the questionnaire survey and I also agree with the subsequent analysis of the data for the purpose of international research and scientific publication. If I had questions about this envisaged study, they were posed and answered by the researcher to my satisfaction.

I agree with the described collection and processing of the data (age, gender, year of study, type of school form and answers to perspectives on social media).

I had enough time to make a decision and I am ready to participate in the above mentioned study. I know that the participation in the study is voluntary and I can discontinue my participation at any time without giving reasons.

**Section 1**

**Basic information**

1. University of studies
2. Age (use range)
3. Gender (F/M/Other)
4. Year of studies (1/2/3/4/5/More)
5. Type of school where you expect to work (Pre-primary school/Elementary or Primary school/Secondary school/Special education school/Vocational school/Other)
6. How often do you login to the following social media? (check an option for each service)

|  | Daily | Weekly | Monthly | Sporadically | I have never used this social media | I previously used this social media, but no longer do so |
| --- | --- | --- | --- | --- | --- | --- |
| Twitter |  |  |  |  |  |  |
| Facebook |  |  |  |  |  |  |
| Instagram |  |  |  |  |  |  |
| Snapchat |  |  |  |  |  |  |
| Whatsapp |  |  |  |  |  |  |
| Groupme |  |  |  |  |  |  |
| Pinterest |  |  |  |  |  |  |
| Other |  |  |  |  |  |  |

6.1. Which other social media do you use? (short textbox)

7. In which of those social media do you consider yourself as an active user, who contributes with messages and comments (in contrast to a passive user, who only reads what others write)? (check all the boxes that apply) Facebook, Twitter, Instagram, Snapchat, Whatsapp, Pinterest, Other, In none of them

8. Have you ever read the privacy policy of a social media tool that you signed up to use? Yes/No

9. As a student, have you used a specifically educational social media service? (e.g., Edmodo, Schoology)?

**Section 2**

**Use of social media for educator professional learning, development, & community**

Indicate your degree of agreement or disagreement with the following statements

(1. Strongly disagree, 2. Disagree, 3. Neutral, 4. Agree, 5. Strongly agree):

10. The use of social media as a teacher is beneficial for professional growth.

11. Social media facilitates the sharing of resources and ideas among educators.

12. I can receive professional mentorship and support via social media.

13. Social media supports reflective thinking skills (e.g., reflection during educational practices, self-assessment processes)

14. I know educators from face-to-face interactions who use social media in a creative and positive way.

15. I have seen online examples of teachers who use social media to connect and collaborate with other educators.

16. I will use social media in the future for my professional development as teacher.

17. I find the quality of educational resources shared via social media to be acceptable.

18. I find that the educational resources shared via social media are appropriate for use in school.

19. Here you can share an example or pose any comments/questions that you may have regarding the use of social media for educator professional learning, development & community. (textbox)

**Section 3**

**Use of social media in schools with students**

Indicate your degree of agreement or disagreement with the following statements

(1. Strongly disagree, 2. Disagree, 3. Neutral, 4. Agree, 5. Strongly agree):

20. Social media can be used with students in educationally beneficial ways.

21. Students use social media in a distracting way more than for learning aims.

22. I know teachers that use social media with their students in educationally beneficial ways.

23. I know teachers and schools that use social media to share information with their local communities.

24. I know teachers that use social media to communicate with and engage families in the learning process.

25. I am favourable to the use of social media in schools.

26. I believe student data should be anonymised when using social media in schools.

27. Teachers have a responsibility to teach students how to avoid problems associated with social media (e.g., cyberbullying, spamming, phishing).

28. Teachers have a responsibility to teach students how to use social media in positive and educational ways.

29. Teachers have a responsibility to collaborate with families to promote positive and secure educational uses of social media.

30. The use of social media in schools can threaten the privacy of students’ data.

31. Only social media that is designed for educational purposes (e.g., Edmodo) should be used in schools.

32. Here you can share an example or pose any comments that you may have regarding the use of social media in schools with students. (textbox)

**Section 4**

**Data privacy beliefs**

Indicate your degree of agreement or disagreement with the following statements

(1. Strongly disagree, 2. Disagree, 3. Neutral, 4. Agree, 5. Strongly agree):

33. I am aware of national data privacy policies related to the personal use of social media (in the EU: General Data Protection Regulation (GDPR), in the U.S.: COPPA/FERPA).

34. I consider myself familiar with national data privacy policies related to the use of social media by children and young people.

35. I consider myself familiar with national data privacy policies related to the use of social media with didactic purposes in the schools.

36. I am aware of the data privacy policies of different social media services that younger students and I use.

37. Teachers have a responsibility to teach students about data privacy policies and practices.

Do data privacy policies in your country.... (Y/N/ or N/A)

38. allow you to use social media for your own professional purposes?

39. allow teachers to use social media for educational purposes with students?

40. Here you can share an example or pose any comments/questions that you may have regarding data privacy beliefs. (textbox)

**Section 5**

**Concerns/confidence/comfort about social media companies**

Indicate your degree of agreement or disagreement with the following statements

(1. Strongly disagree, 2. Disagree, 3. Neutral, 4. Agree, 5. Strongly agree, N/A: I do not know how data is being used by social media services):

41. I am comfortable with how social media companies use my data.

42. I am comfortable with how social media companies use students’ data.

43. I am confident that my country’s government can effectively regulate social media companies’ use of my data.

44. I am comfortable with how Facebook uses my data.

45. I am comfortable with how Twitter uses my data.

46. I am comfortable with how Instagram uses my data.

47. I am comfortable with how Snapchat uses my data.

48. I am comfortable with how Whatsapp uses my data.

49. I am comfortable with how educational social media (e.g., Edmodo, Schoology) use my data.

50. Here you can share an example or pose any comments/questions that you may have regarding concerns or confidence about social media companies. (textbox)

**Appendix 2**

Differences in levels of agreement across university groups on items related to data privacy

|  | **Survey item^a^** | **χ2(3)** | **p =** | **Post hoc pairwise comparisons with adjusted significant differences^b^** | **Post hoc adj. sigs^c^** |
| --- | --- | --- | --- | --- | --- |
| 1 | The use of social media in schools can threaten the privacy of students’ data. | 7.171 | .067 | n/a | n/a |
| 2 | I am aware of national data privacy policies related to the personal use of social media (COPPA/FERPA). | 7.563 | .056 | n/a | n/a |
| 3 | I consider myself familiar with national data privacy policies related to the use of social media by children and young people. | 6.353 | .096 | n/a | n/a |
| 4 | I consider myself familiar with national data privacy policies related to the use of social media with didactic purposes in the schools. | 9.977 | **.019** | SPA > USA | .048 |
| 5 | I am aware of the data privacy policies of different social media services that younger students and I use. | 2.280 | .516 | n/a | n/a |
| 6 | Teachers have a responsibility to teach students about data privacy policies and practices. | 16.098 | **.001** | NZL > USA  NZL > GER | .001  .004 |
| 7 | I am comfortable with how social media companies use my data. | 14.493 | **.002** | USA > GER | .004 |
| 8 | I am comfortable with how social media companies use students’ data. | 8.080 | **.044** | USA > NZL | .049 |
| 9 | I am confident that my country’s government can effectively regulate social media companies’ use of my data. | **10.563** | **.014** | SPA > GER | .025 |
| 10 | I am comfortable with how Facebook uses my data. | **31.148** | **< .001** | USA > GER  SPA > GER | < .001  .002 |
| 11 | I am comfortable with how Twitter uses my data. | **11.342** | **.010** | none | n/a |
| 12 | I am comfortable with how Instagram uses my data. | **20.278** | **< .001** | USA > GER | < .001 |
| 13 | I am comfortable with how Snapchat uses my data. | **11.321** | **.010** | none | n/a |
| 14 | I am comfortable with how WhatsApp uses my data. | **11.492** | **.009** | none | n/a |

^a^For each survey item listed in this table, the null hypothesis tested was that the distribution of Indicate your degree of agreement or disagreement with the following statement for [survey item] was the same across categories of university.

^b^There were six pairwise comparisons for each post hoc test, and only comparisons that resulted in adjusted significances < .05 are included in this table.

^c^Asymptotic significances (2-sided tests) are displayed. Post hoc significance values have been adjusted by the Bonferroni correction for multiple tests.
